# Wave Manipulations by Coherent Perfect Channeling

**DOI:** 10.1038/s41598-017-14422-9

**Published:** 2017-10-24

**Authors:** Xiaonan Zhang, Chong Meng, Z. Yang

**Affiliations:** Department of Physics, the Hong Kong University of Science and Technology Clearwater Bay, Kowloon, Hong Kong, China

## Abstract

We show that through the wave energy conserved and reversible process of coherent interactions of scalar waves in a multi-channel system joint by a common junction, it is possible to generate outgoing waves only in certain channels by controlling the incoming waves. We refer to such processes as coherent perfect channeling (CPC). As two particular examples, we report experimental and theoretical investigations of CPC with two incoming coherent waves in three and four-channel waveguides that are completely channeled into one or two other waveguides mediated by a deep subwavelength dimension scatterer at the common junction. Two such scatterers are discovered, one confirmed by experiments and the other predicted by theory, and their scattering matrices are constructed. Scatterers with other CPC scattering matrices are explored, and preliminary investigations of their properties are conducted. The scattering matrix formulism makes it possible to extend the domain of CPC to other scalar waves, such as electromagnetic waves and quantum wavefunctions.

## Introduction

Strong interaction between two coherent waves can take place when mediated by a suitable scatterer in deep subwavelength dimension, and lead to extraordinary effects. In coherent perfect absorption (CPA) of electromagnetic waves, which was theoretically predicted as the reverse process of lasing^1^, the scatterer is an absorber with suitable transmission, reflection, and absorption coefficients^1^. It was confirmed experimentally first in a silicon cavity^2^, and then in dielectric metasurfaces^3^. The process in a strongly interacting system was also observed^4^. In coherent perfect rotation^5^, which refers to reversible processes transferring any fixed input polarization state of coherent counter propagating light waves completely into its orthogonal polarization, the mediator is a dielectric Faraday rotator. Bending of light beams to the wrong side of the normal direction of a corrugated free-standing metal film under bidirectional coherent illumination was also predicted^6^. Perfect and broadband absorption could be realized by critical coupling in subwavelength resonators^7^, and by asymmetric resonant scatterers^8,9^. Total sound transmission without reflection through ultranarrow channels on a rigid panel mediated by resonators with nearly zero dynamic mass density was first theoretically predicted^10^ and then experimentally demonstrated^11^. In CPA of acoustics waves in waveguides^12^, the scatterers were made of decorated membrane resonators (DMR’s)^13^ and hybrid membrane resonators (HMR’s)^9,14^. The highest ratio of incoming wave intensity over the outgoing one obtained at optimum acoustic CPA conditions was 975 times, and the phase sensitivity of the process was demonstrated. The transmission and reflection condition required for acoustic CPA could also be fulfilled by nonlinearity of a Helmholtz resonator (HR)^15^. In many applications, such as all-wave interference logic gates and integrated optical circuits^16–18^, waveguide modes rather than open space ones are preferred. Plasmon-based interferometric logic operations in silver nanowire networks^16^ and nanoscale plasmonic slot were demonstrated^17^. All-optical AND, XOR, and NOT logic gates based on Y-branch were also investigated in photonic crystal waveguide^18^. Likewise, acoustic logic gates and Boolean operations based on self-collimating acoustic beams^19^ and density-near-zero metamaterials^20^ were investigated. However, the logic operations are not ‘perfect’ in that there are always residue reflection in the input channels and spurious scattering waves at the gate junction. Such imperfections could be eliminated if by controlled coherent interaction of incoming waves with a suitable scatterer at the junction, outgoing waves are generated only in certain channels, while being totally absent in others. If total wave energy is conserved in the process, then the time reversal symmetry is preserved. We refer to such processes as coherent perfect channeling (CPC).

We first report the experimental demonstration of CPC of acoustic waves in the simplest but none-trivial system, namely the three-port T-configuration waveguide system. When two counter propagating waves with the same phase and amplitude are incident from the two main waveguides, all the incident waves are turned, or channeled, into the side branch waveguide with only 3% of the wave energy dissipated in the process. In the reverse process, which is ensured by time reversal symmetry and confirmed by numerical simulations, an input wave in the side branch is totally split into two coherent outputs in the two main waveguides with perfectly matched phase and amplitude. On the other hand, no wave is turned into the side branch when the phase difference of the two incoming waves in the main waveguide is 180°. Theoretical studies also predict similar phenomenon in four-port X-configuration waveguide system, in which incoming waves in any two waveguides are perfectly channeled into the other two channels. Scattering matrices for the three-port and four-port configurations are then formulated, which preserve time reversal symmetry and total wave flux. This implies that the findings in acoustic waves could be generalized to other scalar waves, such as electromagnetic waves, quantum wavefunctions, and spin waves^21^, where time reversal symmetry and linear superposition principle are upheld. The potential of CPC processes in a wide range of applications, including all-wave logic gates, precision interferometry, highly coherent source arrays, and other coherent perfect manipulation of waves, are discussed.

## Method

**Figure 1:**
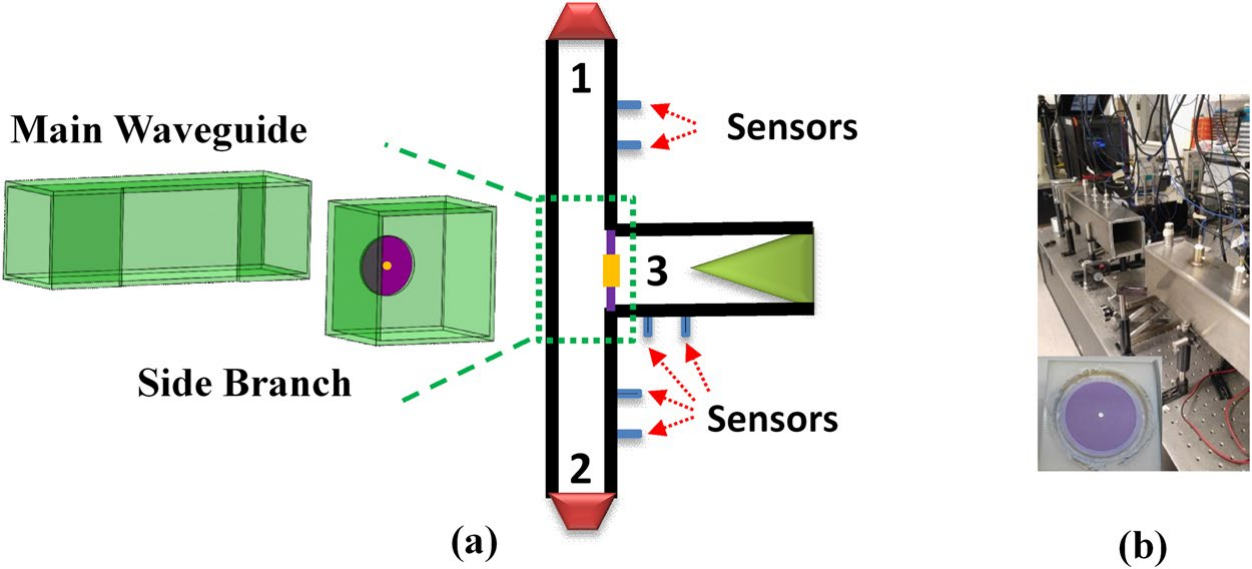
(**a**) The schematics of the experimental setup. (**b**) Photo of the main waveguide. The insert is a photo of a DMR.

The experimental setup for the three-port CPC is schematically shown in Fig. 1(a). The vertical main waveguide (port-1 and -2) is the same as the one used in earlier CPA experiments^9^. Out of convenience, the inner cross section of both ports is 90 mm × 90 mm. A third waveguide (side branch) with twice the inner cross section area (127 mm × 127 mm) is connected at the T-junction to the main waveguide. As will be discussed below, such choice of area ratio between the main waveguide and the side branch is necessary for the type of scatterer used for CPC, which is a DMR mounted at the entrance of the side branch, with a rubber membrane 61 mm in diameter and decorated by a central platelet 5.5 mm in diameter and 30 mg in mass. The rest of the 127 mm × 127 mm entrance cross section area is filled by hard plastic plate about 5 mm in thickness. The sensor near the entrance in the side branch is 100 mm from the entrance plane. The second sensor is 100 mm behind the first sensor. The far end of the side branch is terminated by an anechoic sponge wedge. Similar to our earlier work^12^, from the readings of the six sensors, we obtain the amplitudes and phases of the incoming and outgoing acoustic waves in the three sections of the waveguide system. A photo of the main waveguide is shown in Fig. 1(b). The insert is a photo of a DMR similar to the one used in the experiments. Viewed in the main waveguides, the side branch with the DMR serves as a monopole resonator similar to an HMR mounted on the sidewall of the main waveguide^12^. Numerical simulations using the COMSOL MultiPhysics software package were carried out to verify the underline mechanism for CPC in various configurations. Actual device structures parameters were used in the simulations when they were applicable. The mass density, Poisson’s ratio, Young’s modulus, and the pre-stress of the membrane were 940 kg/m^3^, 0.49, 2 × 10^5^, and 0.5 MPa, respectively. The dissipation is introduced in the form of an imaginary part in the tension that is about 1% of the real part, similar to previous works^13–15^.

### Three-port T-configuration CPC

Consider a three-port acoustic waveguide system shown in Fig. 1(a) with port-1 and port-2 forming the main waveguide, a side branch port-3, and a scatterer at the junction. In general the incoming waves with amplitudes ${({I}_{1}{I}_{2}{I}_{3})}^{T}$ from port-1, -2, and -3 will be scattered at the junction, resulting in outgoing waves with amplitudes ${({O}_{1}{O}_{2}{O}_{3})}^{T}$ towards port-1, -2, and -3. We are looking for conditions for monopolar CPC symbolically denoted as ${(110)}^{T}\to {(00{O}_{3})}^{T}$ to present the process that for two waves of the same amplitude and phase coming from port-1 and port-2, respectively, the only outgoing wave would be towards port-3. If we further require that there is no wave energy loss in the process, then time reversal symmetry is preserved, and the reverse monopolar CPC process denoted as ${(00{O}_{3})}^{T}\to {(110)}^{T}$ should also be allowed. In this process, the incoming wave from port-3 is split into two perfectly matched outgoing waves, one to port-1 and the other to port-2, but without reflected wave by the junction towards port-3. Furthermore, we hope that the dipolar CPC process denoted as ${(1-10)}^{T}\to {(-110)}^{T}$ would also be possible. The monopolar and dipolar CPC are similar to the corresponding ones in CPA if viewed only within the main waveguide, while the reverse monopolar CPC is analogous to lasing or reverse CPA. A CPA scatterer dissipates wave energy, but a CPC scatterer only re-emits acoustic waves while preserves the total wave energy. Therefore, all CPC processes could be carried out without conversion of vibrational energy into any other forms of energy, leading to preservation of time reversal symmetry. CPA processes are irreversible, because they must involve conversion of acoustic energy into other form of energy, such as thermal energy.

Several conditions must be met for the realization of the monopolar CPC process envisioned above. Consider first a single input from port-1 or port-2. The incoming wave will in general be divided into three outgoing waves, namely the reflected wave back to port-1 denoted by the reflection coefficient *r*, the outgoing wave towards port-2 denoted by the transmission coefficient *t*, and the outgoing wave towards port-3 denoted by the turning coefficient *τ*. Consider only the waves in the main waveguide without input from port-3, we then have $$(\begin{array}{c}{O}_{1}\\ {O}_{2}\end{array})=(\begin{array}{cc}r & t\\ t & r\end{array})(\begin{array}{c}{I}_{1}\\ {I}_{2}\end{array})$$. To realize monopolar CPC denoted by $$(\begin{array}{c}0\\ 0\end{array})=(\begin{array}{cc}r & t\\ t & r\end{array})(\begin{array}{c}1\\ 1\end{array})$$, we must have *t* = −*r*, namely the amplitudes of the two coefficients must be the same, and their phase difference must be 180°. For subwavelength scale devices with monopolar symmetry, the amplitude of *t* and *r* must be 0.5^22^. So the first condition for monopolar CPC is that *t* = −*r* = 0.5. Under such condition, $$(\begin{array}{c}-1\\ 1\end{array})=\frac{1}{2}(\begin{array}{cc}-1 & 1\\ 1 & -1\end{array})(\begin{array}{c}1\\ -1\end{array})$$, i. e., the conditions for dipolar CPC are automatically fulfilled. This is similar to CPA when viewed only within the main waveguide.

For a monopole scatterer like the HMR or a HR mounted on the sidewall of a waveguide, the acoustic transmission in the waveguide is usually large and the reflection is small. The phase of the transmission coefficient also remains small, as the waveguide is almost completely clear for wave propagation. At the monopolar resonant frequency a soft boundary with nearly zero impedance is created by the scatterer, and the phase of the reflection coefficient becomes 180°. Therefore, only at the monopolar resonance of the scatterer will the *r* and *t* phase difference condition be satisfied. By carefully adjusting the mass of the platelet mounted on the membrane, we can tune both the amplitudes of *t* and *r* to 0.5. Let the surface averaged air speed and pressure near the junction, and the cross section area in port-1 be ${v}_{1}$, ${p}_{1}$, and ${S}_{1}$. Those in port-3 are ${v}_{3}$, ${p}_{3}$, and ${S}_{3}$. The continuity condition implies that $2{v}_{1}{S}_{1}={v}_{3}{S}_{3}$. At resonance the surface impedance of the DMR is zero^10,11,13^, so ${p}_{1}={p}_{3}$. Since there is only one incoming wave each in port-1 and port-2, and one outgoing wave in port-3, we must have ${p}_{1}/{v}_{1}={Z}_{0}={p}_{3}/{v}_{3}$, where *Z*_0_ is the air impedance. We then obtain $2{S}_{1}={S}_{3}$, and ${v}_{1}={v}_{3}$. Therefore, the second condition for the monopolar CPC is that the cross section area of the side branch must be two times of that of the main waveguide, and CPC only occurs at the resonant frequency of the monopolar scatterer.

The third condition for monopolar CPC is that the amplitude of the turning coefficient must be 0.5. This is dictated by wave energy conservation, such that under single input from port-1 or port-2, $|r{|}^{2}+|t{|}^{2}+2|\tau {|}^{2}=1$ is satisfied. As will be shown below, the conditions set for monopolar CPC are also the necessary and sufficient ones for reverse monopolar CPC and dipolar CPC.

**Figure 2:**
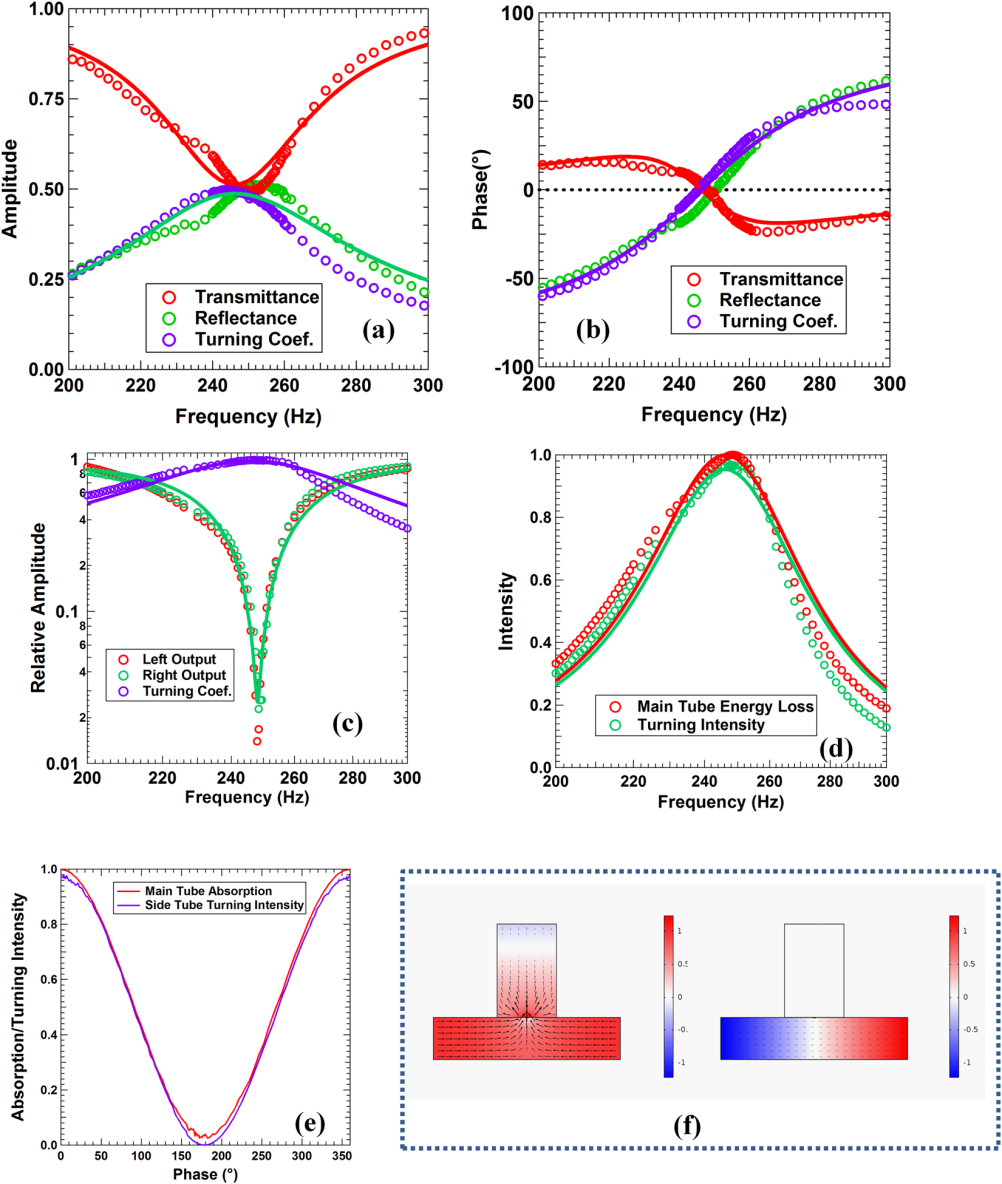
In (**a**) through (**d**), the circles are experimental data and the solid curves are theoretical simulation results. (**a**) The one-side incidence transmission (red), reflection (green) coefficients in the main waveguides and the turning coefficient (purple) from the main waveguides into the side branch. (**b**) The phase spectra of the transmission (red), reflection (green), and the turning coefficient (purple). For clarity, the reflection phase has been shifted downwards by 180°. (**c**) The CPC spectra consisting of the amplitudes of the outgoing waves (red and green) in the main waveguides and the turning coefficient into the side branch (purple). (**d**) The apparent CPA intensity (red) and the turning intensity (green) spectra. (**e**) The experimental apparent CPA intensity and the turning intensity as a function of the phase difference between the two incoming waves in the main waveguide. (**f**) The pressure and air velocity fields at zero phase difference (left) and 180° out of phase (right) between the two incoming waves.

Similar to the procedure to achieve CPA, the transmission *t* and the reflection *r* of the junction under one side incidence (say from port-1) in the main waveguide were measured first. Figure 2(a) shows the experimental transmission (red circles) and reflection (green circles) spectra together with the simulation results (appropriately colored solid curves). At 248.3 Hz the transmission coefficient is 0.496 and the reflection coefficient is 0.498. Both are very close to the critical value of 0.5 for CPA. This is different from extraordinary transmission through narrow channels, where transmission is 100% and reflection is zero^10,11^. The phase of the reflection is 176.2° while that of the transmission is −1.3°, as shown in Fig. 2(b) by the corresponding red and green circles together with the simulation results in solid curves of appropriate colors. The phase difference deviates significantly from 180° at off-resonant frequencies, so at these frequencies the outgoing waves in the main waveguide cannot be fully eliminated when there is an incoming wave from both port-1 and port-2. The seemingly lost wave energy in the main waveguide, however, was actually channeled to the side branch, as the outgoing wave amplitude in the side branch (the turning coefficient *τ*) reached 0.49 in the meantime, as shown in Fig. 2(a) as the purple circles. Total wave energy was conserved as ${r}^{2}+{t}^{2}+2{\tau }^{2}$ is very close to 1. In the theoretical simulations, the turning coefficient nearly coincides with the reflection because both waves are emitted by the DMR, so it is not shown in Fig. 2(a),(b). The transmission and reflection seen in the main waveguides are therefore identical to those for a monopole CPA^12^.

Figure 2(c) shows the apparent CPA seen in the main waveguide. At 248.2 Hz, the experimental outgoing wave amplitudes (red circles for the left outgoing waves and green circles for the right outgoing waves) drop to 1.7% and 2.3%, respectively, of the incoming ones emitted by the left and the right speakers. The theoretical amplitudes for the left outgoing and the right outgoing waves are identical, so only one of them is shown as the solid green curve in the figure. The amplitudes of the incoming waves (not shown in the figure) remain nearly 1 in the entire frequency range of interest. The maximum intensity ratio of the incoming waves over the outgoing ones is over 2400 times, which is significantly higher than the best CPA scatterer reported earlier^12^.

What is truly different from real CPA is that the outgoing wave amplitude in the side branch reaches maximum of 0.99 at the apparent CPA frequency of 248.2 Hz. Nearly all the incoming wave energy in the main waveguides is actually channeled into the outgoing wave in the side branch. This is further supported by the measured apparent main waveguide loss coefficient shown as purple circles in Fig. 2(c) and red circles in Fig. 2(d) and the turning coefficient into the side branch (green circles) shown in Fig. 2(d). The two coefficients are nearly the same, showing that the loss in the main waveguide has been mostly channeled into the outgoing wave in the side branch. At the optimum frequency, nearly 97% of the energy is channeled into the side branch, while 3% of the energy is dissipated by the DMR. The process is therefore a vivid example of CPC.

Another essential feature in the two-wave coherent interplay is the dependence of the outcome, being absorption, rotation, or in this case channeling, on the relative phase $\varphi$ of the incoming waves in the main waveguides. Indeed, as shown in Fig. 2(e), both the experimental main waveguide absorption and the turning coefficient exhibit ${\cos }^{2}(\varphi /2)$ dependence, which are similar to monopolar CPA^12^. The minimum turning coefficient is 3.7 × 10^−5^ reached at $\varphi$ = 179.9°, which is in fact what the dipolar CPC calls for. The contrast ratio of the turning coefficient at maximum of 0.97 over the minimum is therefore 2.6 × 10^4^ times, or 44 dB. If used as a phase sensitive detector at this optimum phase difference, the intensity would change by nearly 7 times per degree, 26 times more sensitive than the best one reported earlier^12^.

As shown in Fig. 2 (a) through (d), the theoretical predictions (solid curves) agree well with the general features of the experimental results. We therefore conducted theoretical studies to explore further the properties of CPC. Shown in Fig. 2(f) are the pressure and air velocity fields obtained from the simulations at optimum CPC in-phase condition (left) and out-of-phase condition (right). It is seen that at monopolar CPC the incoming waves in the main waveguides are channeled completely into the side branch. However, unlike the real CPA case for the side mounted HMR^12^ in which the membrane vibration is enhanced by nearly 70 times for wave energy dissipation, here the air velocity is enhanced by about 14 times, resulting in the real dissipation of only 3%. At CPC the platelet is vibrating in unison with the membrane, similar to that of the first eigenmode of the DMR at 259.8 Hz. At 180° off phase for dipolar CPC, the waves mostly stay in the main waveguide with little channeling into the side branch, and the membrane remains almost motionless.

The near-zero dissipation implies that CPC obeys time reversal symmetry, which dictates that the reverse of CPC can also take place. Indeed, our simulations show that at the CPC frequency, the incoming wave in the side branch will almost totally split into two identical outgoing ones in the main waveguides, with little reflection back to the side branch. The frequency dependence of the reflection and the turning coefficient in the reverse monopolar CPC resemble closely the spectra in Fig. 2(c), with the reflection following the green curve (monopolar CPC main waveguide outgoing waves), and the turning coefficient following the purple curve (monopolar CPC turning coefficient). Such process is somewhat like the extraordinary transmission through narrow channels with resonators of zero dynamic mass density^10,11^. But here it occurs only for incoming wave from the side branch.

### Scattering Matrix for T-configuration CPC

In order to conceptualize the CPC process, we analyze the process in terms of the scattering matrix of a scatterer at the junction. Consider a *N*-port waveguide system with cross section areas *S*_1_, *S*_2_,.., *S*_*N*_, intersecting at a common junction where a scatterer is located. The incoming scalar waves and the outgoing ones are related by the *N* × *N* scattering matrix of the scatterer at the junction given by ${({O}_{1}{O}_{2}\ldots {O}_{N})}^{T}={\hat{M}}_{N}{({I}_{1}{I}_{2}\ldots {I}_{N})}^{T}$. If the net wave flux is conserved in the process, then

1 $$\sum _{i=1}^{N}{S}_{i}|{I}_{i}{|}^{2}=\sum _{i=1}^{N}{S}_{i}|{O}_{i}{|}^{2}$$

The process must obey time reversal symmetry as dictated by the wave equation, if net flux is conserved. In the cases where all the waveguides contain the same medium, we then must also have ${({I}_{1}{I}_{2}\ldots {I}_{N})}^{T}\,=\,{\hat{M}}_{N}{({O}_{1}{O}_{2}\ldots {O}_{N})}^{T}$. This leads to ${\hat{M}}_{N}\cdot {\hat{M}}_{N}={\hat{I}}_{0}$ the unity matrix. The scattering matrix may not be symmetric because of the cross section area differences between different waveguides. The two-port monopole CPA process is irreversible, as the matrix $${\hat{M}}_{2}=\frac{1}{2}(\begin{array}{cc}1 & -1\\ -1 & 1\end{array})$$ does not satisfy the ${\hat{M}}_{2}\cdot {\hat{M}}_{2}={\hat{I}}_{0}$ condition. It is expected because wave energy is dissipated in the process, and time reversal symmetry is broken.

The scattering matrix for the T-configuration CPC presented above can be expressed as $${\hat{M}}_{3}=(\begin{array}{ccc}r & t & \tau \text{'}\\ t & r & \tau \text{'}\\ \tau  & \tau  & r\text{'}\end{array})=$$ $$\frac{1}{2}(\begin{array}{ccc}-1 & 1 & 2\\ 1 & -1 & 2\\ 1 & 1 & 0\end{array})$$. One can verify by simple algebra that $${\hat{M}}_{3}\cdot {\hat{M}}_{3}=(\begin{array}{ccc}1 & 0 & 0\\ 0 & 1 & 0\\ 0 & 0 & 1\end{array})$$, and for any incoming waves ${(abc)}^{T}$, the flux of both the outgoing waves ${P}_{O}$ and incoming waves ${P}_{I}$ are equal to ${a}^{2}+{b}^{2}+2{c}^{2}$. The third channel must be twice in area of the other two, as has been shown above. The matrix implies the following. In the main waveguide connecting port-1 and port-2, the reflection to an incoming wave from port-1 or port-2 at the junction is −0.5, the transmission to port-2 or port-1 is 0.5, and the turning coefficient into the side branch to port-3 is also 0.5. When two identical waves are coming from port-1 and port-2, which is denoted as $\hat{I}={(110)}^{T}$, the outgoing waves are $\hat{O}={(001)}^{T}$, which is the monopolar CPC process. For an incoming wave in the side branch from port-3 denoted as, $\hat{I}={(001)}^{T}$, the reflection at the junction is 0, and the turning coefficient is 1, so $\hat{O}={(110)}^{T}$. This is the reverse monopolar CPC process. For dipolar CPC input denoted as $\hat{I}={(1-10)}^{T}$, the output given by the scattering matrix is $\hat{O}={(-110)}^{T}$, as expected. All these characteristics of the scattering matrix agree well with the experimental results and theoretical simulations shown in Fig. 2.

### Four-port X-configuration CPC

**Figure 3:**
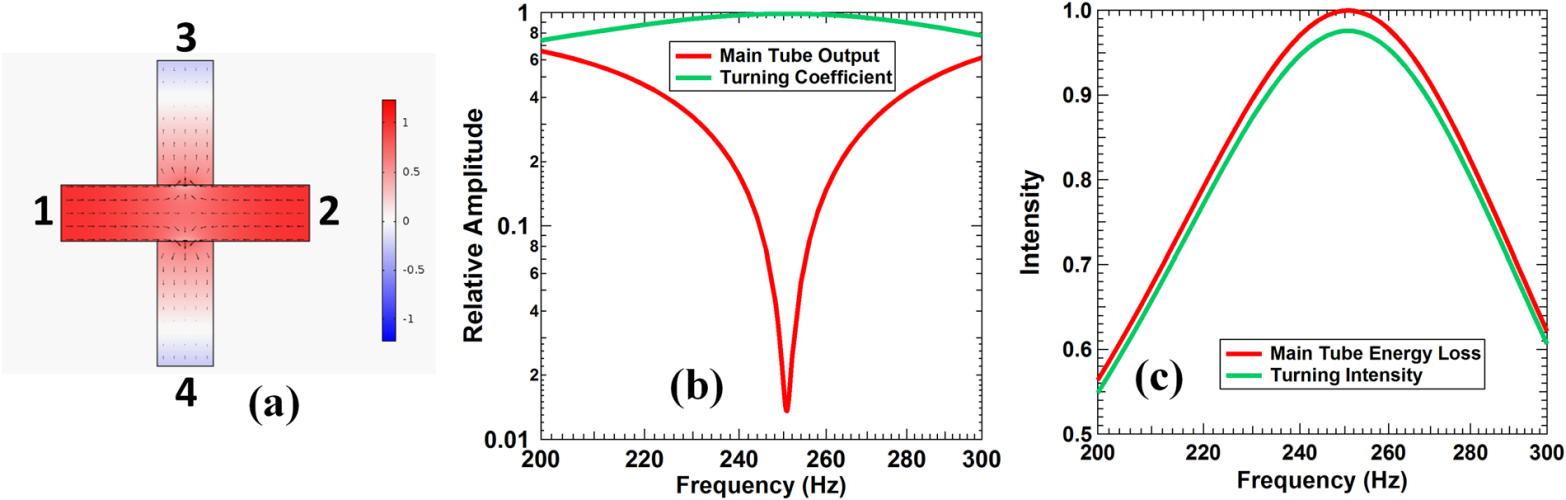
The simulation results of an X-configuration CPC process. (**a**) The pressure and air velocity fields at CPC frequency. A DMR is mounted at the entrances of port-3 and port-4. (**b**) The amplitude of the outgoing waves (red) and the turning amplitude (green) as a function of frequency. (**c**) The main waveguide apparent CPA intensity (red) and the turning intensity (green).

We now extend CPC to four-port X-configuration systems shown in Fig. 3(a). Using similar arguments presented above for the three-port system, if out of convenience we assume that port-1 and port-2 are of the same cross section area, and port-3 and port-4 are of the same cross section area, then all ports must have the same cross section area. A type of scatterers have been discovered through numerical simulations, which have the following scattering matrix

2 $${\hat{M}}_{4}=(\begin{array}{cccc}r & t & \tau  & \tau \\ t & r & \tau  & \tau \\ \tau  & \tau  & r\text{'} & t\text{'}\\ \tau  & \tau  & t\text{'} & r\text{'}\end{array})=\frac{1}{2}(\begin{array}{cccc}-1 & 1 & 1 & 1\\ 1 & -1 & 1 & 1\\ 1 & 1 & -1 & 1\\ 1 & 1 & 1 & -1\end{array})$$

Similar to the T-configuration, for a single input from port-1, the reflection back to port-1 at the junction is *r*, the transmission inward port-2 is *t*, and the turning coefficient to port-3 and port-4 is $\tau$. The same is true for input from port-2. The corresponding ones for an input from port-3 or port-4 are *r′*, *t′*, and $\tau$. It can be verified by simple algebra that ${\hat{M}}_{4}$ preserves total wave flux and time reversal symmetry. Such scatterers could be realized, as predicted by simulations, by mounting a DMR at the junction entrance of each secondary waveguide-3 and -4, as shown in Fig. 3(a). In the present case, the two DMR’s are the same as the one for the T-configuration CPC shown in Fig. 1. At 250.8 Hz, which is slightly different from the T-configuration CPC frequency because of the change in geometry, the reflection for an incoming wave from any port is −0.5, and the transmission and the turning coefficients into the three other waveguides are 0.5. Therefore, viewed either through the horizontal waveguides (waveguide-1 and -2) or the vertical waveguides (waveguide-3 and -4), the junction looks like a CPA monopole^12^. The difference is that the lost wave energy in the horizontal waveguides is channeled equally into the perpendicular waveguides, instead of being dissipated, as shown in Fig. 3(a). Likewise, due to time reversal symmetry, identical incoming waves in waveguide-3 and -4 will be channeled completely and equally into waveguide-1 and -2, as verified by simulations. In fact, as dictated by the matrix in Eq. (2) and verified by simulations, identical incoming waves from any pairs of the four ports will be channeled completely and equally towards the other two ports. As shown in Fig. 3(b),(c), the simulation results for the outgoing wave amplitudes and the turning coefficient are almost the same as the corresponding ones in the T-configuration. The amplitudes of the outgoing waves in the horizontal waveguides (red curve in Fig. 3(b)) drop to a minimum of nearly 1% at 250.8 Hz, while the amplitudes of the outgoing waves (green curve) in the vertical waveguides reach maximum. As indicated in Fig. 3(c), the apparent energy loss in the horizontal waveguides is almost the same as the outgoing wave intensity in the vertical ones. The actual dissipation is about 3% at the maximum CPC frequency. As is in the T-configuration, dipolar CPC occurs when the relative phase difference between the two incoming waves is 180°.

The T-configuration in Fig. 1 can be regarded as a special case of the X-configuration with waveguide-3 and -4 bundled together. That is, if one assumes that the waves in waveguide-3 and -4 are always identical, then one can reproduce CPC in the T-configuration. The zero reflection at the T-junction for the side branch is actually a manifestation of the CPC process for the two identical incoming waves in waveguide-3 and -4, respectively. This is consistent with the fact that the cross section area of the side branch in the T-configuration must be twice the ones of the main waveguides.

### Other Possible CPC Matrices

There are other possible CPC matrices for the X-configurations. The identification of these matrices could expand the scope of CPC, and provide guidance for the designs of suitable scatterers. The matrix in Eq. (2) can be expressed as $${\hat{M}}_{4}=(\begin{array}{cc}\hat{A} & \hat{B}\\ \hat{B} & \hat{A}\end{array})$$, with $$\hat{A}=\frac{1}{2}(\begin{array}{cc}-1 & 1\\ 1 & -1\end{array})$$ and $$\hat{B}=\frac{1}{2}(\begin{array}{cc}1 & 1\\ 1 & 1\end{array})$$. It is straightforward to show that $\hat{A}\cdot \hat{A}=-\hat{A}$, $\hat{B}\cdot \hat{B}=\hat{B}$, and $\hat{A}\cdot \hat{B}=\hat{B}\cdot \hat{A}=0$. Two other possible monopole CPC matrices are $$(\begin{array}{cc}-\hat{A} & \hat{B}\\ \hat{B} & -\hat{A}\end{array})$$ and $$(\begin{array}{cc}\hat{A} & \hat{B}\\ \hat{B} & -\hat{A}\end{array})$$. It is straightforward to show that they preserve total wave flux and time reversal symmetry. The first one is similar to that in Eq. (2), in that identical incoming waves from any pairs of the four ports will be channeled completely towards the other two ports.

The second one would produce what we refer to as the ‘traffic light effect’. Consider equal inputs from port-1 and -3, $\hat{I}={(1010)}^{T}$, the output is $\hat{O}={(0110)}^{T}$. The incoming wave in waveguide-3 is totally reflected as if blocked by a hard wall, while the incoming wave in waveguide-1 totally passes through the junction to waveguide-2. This is like the wave in waveguide-3 ‘sees’ a ‘red light’ at the junction, while the wave in waveguide-1 ‘sees’ a green light and passes unimpededly to waveguide-2. The existence of waveguide-4 does not seem to matter, but its presence actually provides the scattering to channel the waves. In the second case, when the two input waves are in the opposite phase form, $$\hat{I}={(\begin{array}{cccc}-1 & 0 & 1 & 0\end{array})}^{T}$$, then the outgoing waves are $\hat{O}={(100-1)}^{T}$. Now the wave in waveguide-1 ‘sees’ a ‘red light’ and is reflected by a perfectly soft boundary, and the wave in waveguide-3 totally passes through to waveguide-4. Waveguide-2 does not seem to exist, but its presence actually provides the necessary scattering at the junction. Such effect demonstrates that a wave in waveguide-3 or -4 could intersect the waves in waveguide-1 and -2, depending on their relative phase. Likewise, the waves in waveguide-1 and -2 also have similar power in the manipulation of the waves in waveguide-3 and -4.

Another interesting scenario is when waveguide-1 carries two waves of the same amplitude and opposite phase while waveguide-3 carries a wave twice the amplitude, $\hat{I}={(1010)}^{T}+{(-1010)}^{T}$, then the wave in waveguide-1 that is in phase with that in waveguide-3 will pass through to waveguide-2, while the out of phase one is reflected back. Although this seems equivalent to $\hat{I}={(0020)}^{T}$ with no wave in waveguide-1, one could make the distinction by using two sub-waveguides for waveguide-1 that combine only near the junction, each sub-waveguide having only half the cross section area as the other three waveguides. This could be difficult for acoustic waves but is readily available in fiber optics.

Possible dipolar CPC matrices are $$(\begin{array}{cc}\hat{B} & \hat{A}\\ \hat{A} & \hat{B}\end{array})$$, $$(\begin{array}{cc}\hat{B} & -\hat{A}\\ -\hat{A} & \hat{B}\end{array})$$, and $$(\begin{array}{cc}\hat{B} & -\hat{A}\\ \hat{A} & \hat{B}\end{array})$$ that completely channel dipolar inputs, which can be shown in a straightforward way that they preserve total wave flux and time reversal symmetry. As an example, the first dipole scattering matrix will turn dipolar input $\hat{I}={(1-100)}^{T}$ into dipolar output $\hat{O}={(00-11)}^{T}$. The other two have similar effects.

The above scattering matrices derived originally from acoustic wave scatterers could be generalized to other scalar waves, as long as they obey time reversal symmetry and linear superposition principle. As the total flux is conserved, it could ease the difficulties in finding the right scatterers in waves other than acoustic ones, especially for quantum wavefunctions where scattering potentials that conserve particle numbers are much more common than the ones that do not.

### Discussions and Summary

The CPC process could significantly extend the coherent perfect manipulations of scalar waves, and spin off to CPX, where ‘X’ stands for any conceivable wave manipulations. Any CPX process is effectively divided into two steps. The incoming waves are first coherent perfectly channeled to other channel(s), in which they could then be manipulated. If the manipulation is absorption, the process will then be the same as CPA, although real absorption could take place in a much larger space well separated from the CPC region. With the absorbing core removed from the scatterer, more flexibility in design of null detectors becomes possible^12^. The reverse CPC in T-configuration splits one incident wave into two identical waves without the involvement of real lasing process. If cascaded further down *n*-fold, 2^*n*^ identical waves could be generated with relative ease. The two split waves could also serve as the input for a second CPC, instead of using two conventional sound sources prone to imperfections^12^. Such combination could serve as a high sensitivity interferometer with the incident wave first divided into a perfectly matched pair, and then combined as the inputs for the second CPC for phase sensitive detection.

In the CPC processes total flux is strictly conserved, which leads to total wave energy conservation for acoustic wave CPC. In reality, there is always a little energy dissipation in the vibrating mechanical system. That is the main reason why in the above experiments wave energy is almost, but not strictly, conserved. Total flux conservation implies that the CPC process is the most cascadable in all-wave logic gate operations. It is straightforward to show that the CPC T-configuration can perform all the Boolean operations reported in ref.^18^. The subwavelength nature of the scatterers implies compact and perhaps ultrahigh speed devices if their counterparts in electromagnetic waves and quantum waves could be realized. A network of waveguides having a particular type of scatterer at each junction could have intriguing wave manipulation and logic operation capabilities. The full potential of such networks is yet to be explored.
